# Free-carrier-induced soliton fission unveiled by *in situ* measurements in nanophotonic waveguides

**DOI:** 10.1038/ncomms11332

**Published:** 2016-04-15

**Authors:** Chad Husko, Matthias Wulf, Simon Lefrancois, Sylvain Combrié, Gaëlle Lehoucq, Alfredo De Rossi, Benjamin J. Eggleton, L. Kuipers

**Affiliations:** 1 Centre for Ultrahigh bandwidth Devices for Optical Systems (CUDOS), Institute of Photonics and Optical Science (IPOS), School of Physics, University of Sydney, Sydney, New South Wales 2006, Australia; 2 Center for Nanophotonics, FOM Institute AMOLF, Science Park 104, 1098 XG, Amsterdam, The Netherlands; 3 Thales Research and Technology, 1 Avenue. A. Fresnel, 91767 Palaiseau, France

## Abstract

Solitons are localized waves formed by a balance of focusing and defocusing effects. These nonlinear waves exist in diverse forms of matter yet exhibit similar properties including stability, periodic recurrence and particle-like trajectories. One important property is soliton fission, a process by which an energetic higher-order soliton breaks apart due to dispersive or nonlinear perturbations. Here we demonstrate through both experiment and theory that nonlinear photocarrier generation can induce soliton fission. Using near-field measurements, we directly observe the nonlinear spatial and temporal evolution of optical pulses *in situ* in a nanophotonic semiconductor waveguide. We develop an analytic formalism describing the free-carrier dispersion (FCD) perturbation and show the experiment exceeds the minimum threshold by an order of magnitude. We confirm these observations with a numerical nonlinear Schrödinger equation model. These results provide a fundamental explanation and physical scaling of optical pulse evolution in free-carrier media and could enable improved supercontinuum sources in gas based and integrated semiconductor waveguides.

## Abstract (web summary)

Solitons are nonlinear waves that exist in diverse forms of matter. Here, Husko *et al*. use near-field measurements to observe the spatio-temporal evolution of optical pulses in a nanophotonic semiconductor waveguide, demonstrating that nonlinear photo-carrier generation can induce fission of solitons.

## Graphical abstract

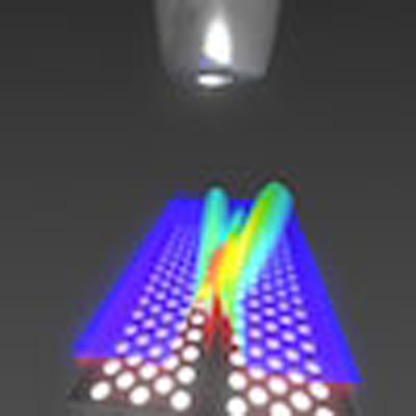

Soliton fission occurs when a fundamental soliton is ejected and temporally separates from a higher-order soliton due to a sufficiently strong perturbation to the system. This behaviour strongly contrasts with the expected periodic recurrence for ideal higher-order solitons^1^. In the optical domain, soliton fission or ‘soliton decay' as it is also known, was first numerically shown to occur due to perturbations of the traditional nonlinear Schrödinger equation including self-steepening (SS), third-order dispersion (TOD) and Raman scattering^2^,^3^,^4^ with experimental demonstrations following soon after^5^,^6^. Since that time, nonlinear optical waveguides have evolved from glass optical fibres to new platforms such as semiconductors^7^ and gas-filled microstructured fibres^8^ where the dominant perturbation is a plasma effect due to nonlinear photogeneration of free electrons or free carriers (electron–hole pairs) similar to light in bulk ionized gases^9^.

The free-carrier plasma modifies the nonlinear pulse evolution with both dispersive (FCD, *n*_FC_) and absorptive (FCA, σ) contributions leading to non-trivial dynamics unavailable in other optical systems. While in the spectral domain optical pulses undergo a spectral blueshift due to FCD^8^,^9^,^10^, in contrast the temporal properties are governed by the dynamic interaction of FCD and dispersion together leading to, for example, nonlinear pulse temporal broadening^11^. These free-carrier effects can also interplay with and modulate the classical soliton evolution. Temporal solitons in free-carrier media have been shown^12^,^13^,^14^ including soliton self-frequency blueshift^15^ and soliton acceleration^16^,^17^. While recent numerical simulations suggest that free carriers could cause soliton fission^15^, both a theoretical formulation and direct temporal measurements establishing a causal link remain open challenges to the field.

Here we provide both an experimental demonstration and a theoretical explanation of the physics underpinning soliton fission induced by a free-carrier perturbation. Using an interferometric near-field scanning optical microscope (NSOM), we observe both the spatial and temporal pulse evolution *in situ* along a semiconductor waveguide. This direct measurement is essential to unraveling the localized nonlinear dynamics in nanophotonic waveguides as traditional cut back methods used for macroscopic devices are impractical at these length scales. From the theoretical side, we derive an analytic formalism to reveal the physical parameters governing the system. With this new formalism we determine a quantitative threshold required to observe soliton fission induced by FCD and show that our experimental conditions exceed the threshold by an order of magnitude. In our experiment, the fission occurs on a length scale as small as 160 μm due to a slow-light enhancement of the optical field in the photonic crystal waveguide (PhCWG) device. This value represents the shortest fission length we could find reported in the literature. We confirm these results with a numerical model based on the generalized nonlinear Schrödinger equation (GNLSE) incorporating the higher-order effects.

## Results

### Near-field measurements of nonlinear pulse propagation

**Figure 1:**
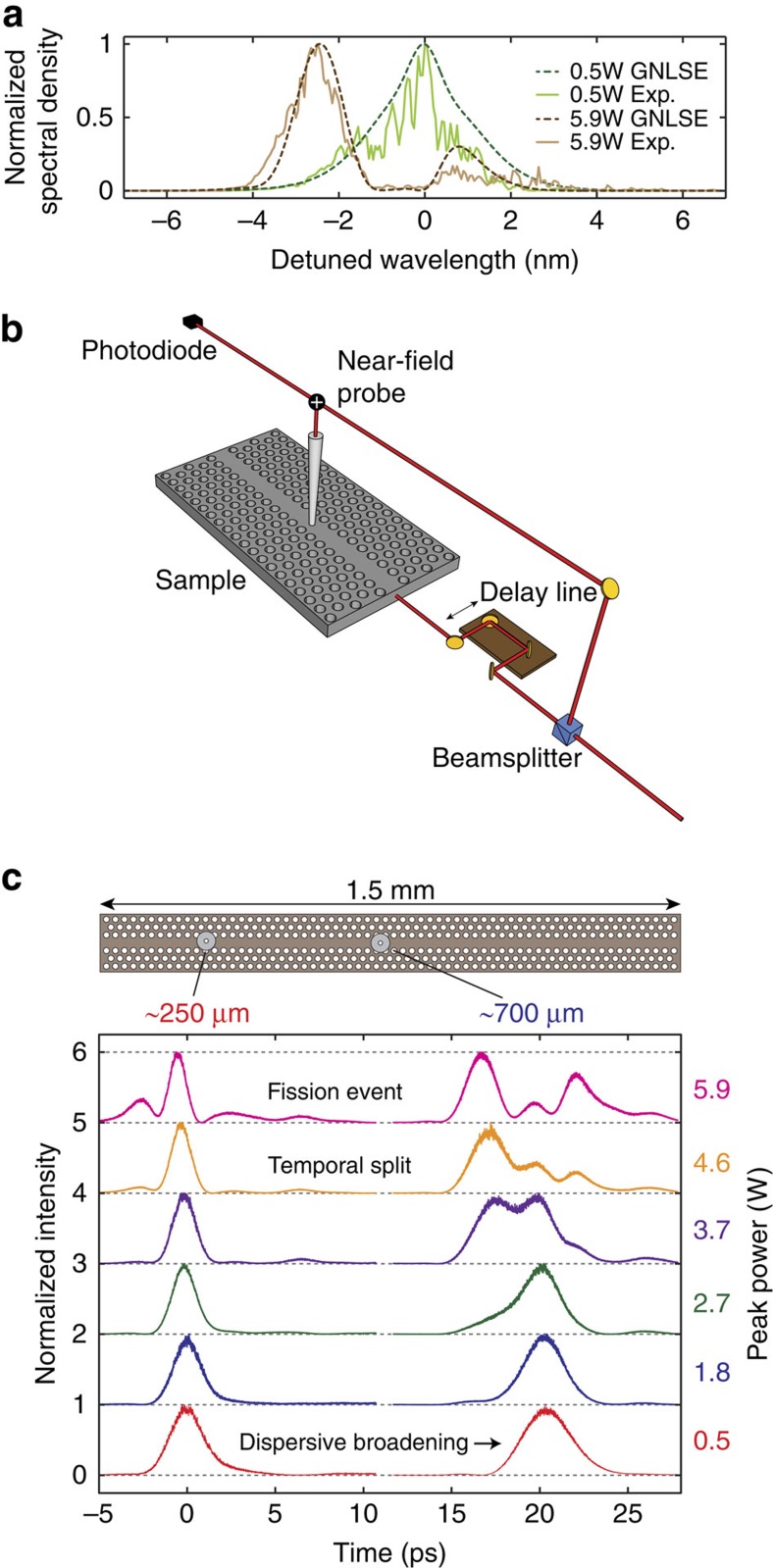
Spectral transmission and time-resolved near-field microscopy of soliton fission. (**a**) Spectral transmission properties of the optical pulse measured at the waveguide output. (**b**) Time-resolved near-field optical microscope (NSOM) apparatus used in the experiment. (**c**) Experimental cross-correlation measurements as a function of power (vertical axis) at two spatial positions along the nanostructured photonic waveguide. It is clear that as the power is increased a break up of the pulse occurs as it propagates.

The structure under study is a two-dimensional PhCWG made of air-holes etched in a GaInP slab (see Methods, Supplementary Note 1 and Supplementary Table 1 for additional details). These structures are known to enhance the nonlinear optical properties due to slow light in the periodic medium^18^. We note the increased group index *n*_g_=15.1 is achieved using the dispersion-engineered design outlined in ref. 19 in a region away from the band edge so as to avoid scattering losses^20^ and minimize TOD^21^. The earliest investigations of nonlinear evolution of optical pulses in PhCWGs examined the pulse spectra after the pulse propagated through the waveguide^22^. Figure 1a shows the measured spectral transmission in our current experiment (solid) at the waveguide output for low and high power levels for the optical pulse of 2.2 ps (*T*_FWHM_, full-width at half-maximum of a hyperbolic secant). Note that the oscillations in the measured spectra arise from disorder in the periodic media^20^. The measured waveguide transmission spectrum is shown as Supplementary Fig. 1. The dashed curves are the result of model calculations detailed below. Spectra measured at different power levels are shown in Supplementary Fig. 2 and described in Supplementary Note 2. We observe a clear spectral blueshift at high power due to FCD^7^, as well as a less intense satellite peak. Such satellite peaks have in the past been attributed to soliton fission in fibres, though no similar observations in semiconductor waveguides have been reported to date.

To determine the origin of the satellite peak it is highly desirable to investigate the pulse evolution as it occurs. Traditionally, this is done through cut back of an optical fibre, wherein measurements are taken at multiple spatial points albeit at the cost of device destruction^23^. This method is impractical for nanoscale devices without high risk of damage to the sample. Fabricating devices of different lengths overcomes this limitation, though with the drawback of device-to-device variation. Non-destructive techniques such as NSOM^24^ or photomodulation spectroscopy^25^ are well suited to evaluate the propagation dynamics of sub-wavelength structures.

Figure 1b illustrates the time-resolved NSOM we used to measure the pulse evolution in the waveguide^21^. With this set-up we are able to measure the temporal dynamics of the propagating pulse inside the waveguide at the position of the near-field probe. In detail, we measure a temporal electric-field cross-correlation between the pulse in the sample and a pulse in the reference branch of the interferometric set-up. Details about the working principle of the NSOM can be found in the Methods. This cross-correlation contains all crucial information about the evolution of the temporal pulse envelope of the electric field. For example, it has been shown that the temporal broadening due to group-velocity dispersion (GVD, β_2_)^26^ or the reshaping due to higher-order dispersion^21^ transfers directly from the temporal pulse envelope to the measured cross-correlation. We utilize this relation between the cross-correlation and temporal pulse envelope to describe the results in this work.

Figure 1c shows a summary of the NSOM measurements of the temporal pulse dynamics as a function of coupled peak power *P*_o_. The horizontal direction indicates two spatial positions that we measured along the device with the near-field probe: 250 μm (left-hand side) to 700 μm (right-hand side). A clear modulation of the pulse dynamics is seen as a function of *P*_o_ in the vertical direction. The soliton number 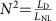 indicates the relative balance of the characteristic length scales for linear dispersion *L*_*D*_ and the nonlinear Kerr effect *L*_NL_ and determines the pulse propagation regime. These lengths will be defined as they are used in the text. In the linear regime the soliton number is *N*=0.5 (*P*_0_=0.5 W) and temporal broadening due to GVD (*β*_2_) dominates the propagation from 250 to 700 μm (ref. 27). This makes sense given the dispersion length of 410 μm 
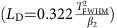
 and a sample length of *L*=1.5 mm. The power-dependent behaviour at the two spatial locations indicates noticeably different evolution patterns. At 250 μm, the pulse narrows with increasing power, indicative of higher-order soliton temporal compression^28^. In contrast, at 700 μm distinct solitons have formed and separated in time for the initially injected *N*≈2 soliton (*P*_0_=5.9 W). This temporal separation is the essence of soliton fission.

To understand the physical origin of this separation from an intuitive perspective we first recall that a change in frequency (spectral shift Ω) in a dispersive medium corresponds to a change in group velocity. This ultimately translates into a shift in temporal position according to the moment evolution equation 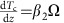 (ref. 29). Since all solitons have anomalous GVD (*β*_2_<0), and here Ω is blueshifted (positive), the result is a temporal advance. This is opposite to the well-known case of solitons in a Raman medium which redshift and therefore slow down^30^,^31^,^32^. In the context of soliton fission, it has been shown that the fissioned constituents have well predicted and very different energies and power levels^33^. As a consequence, the constituent solitons with larger peak power experience a greater self-frequency shift and a larger temporal advance compared with smaller amplitude solitons. Notice in our experiment that the more energetic main soliton is advanced in time due to FCD and dispersion^16^,^29^.

### Confirmation of free-carrier induced fission by modeling

**Figure 2:**
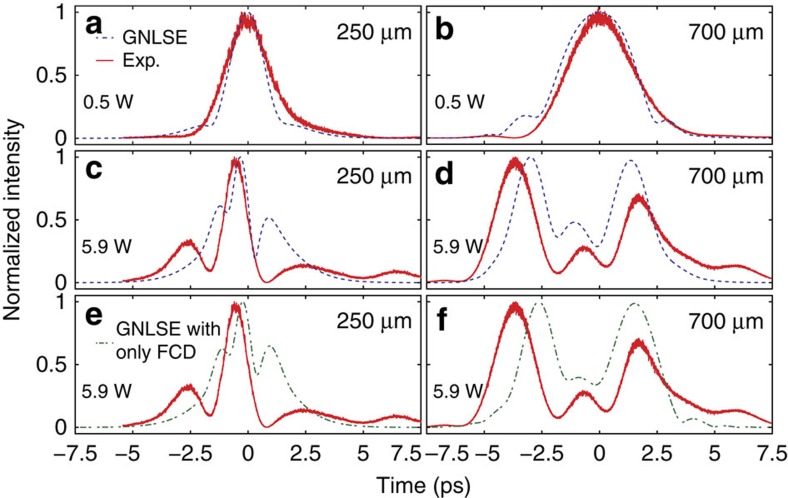
Comparison of experiment and model of the nonlinear pulse propagation. (**a**,**b**) Time-resolved NSOM measurements and GNLSE modelling at a peak power of at a peak power of 0.5 W at a propagation distance of (**a**) 250 μm and (**b**) 700 μm. Temporal broadening of the pulse envelope due to GVD is visible in experiment (red line) and the model (blue line). (**c**,**d**) Same as above with a peak power of 5.9 W. The multiple peaks characteristic of soliton fission are clearly observable in both theory and experiment. To illustrate that the main features observed in the experiment are related to free-carrier generation, (**e**,**f**) compare the experimental results with GNLSE modelling results (green line) taking only the soliton terms and FCD/3PA into account, which still results in a good agreement. Note here we show the cross-correlation of the electric field of the temporal pulse envelope for the modelling as well as the experimental results as defined in the Methods.

The nonlinear pulse propagation in the GaInP semiconductor waveguide can be described by a GNLSE model (Supplementary Note 3). The nonlinear dynamics here are dominated by the *χ*^(3)^ optical Kerr effect (nonlinear parameter *γ*) with free carriers generated by nonlinear three-photon absorption (3PA, *α*_3_) acting as a perturbation in the wide-gap material (*E*_g_=1.9 eV) for our 1,553 nm (∼0.8 eV) pulses (ref. 12). Figure 2a–f show detailed GNLSE modelling (dashed blue and green) with the experimental data (solid red) from Fig. 1 superimposed. In particular, we highlight (a),(b) low and (c),(d) high power at the propagation distance of 250 and 700 μm, respectively. The temporal shapes are in good quantitative agreement and excellent qualitative agreement with the experimental data and capture the essential physics of the nonlinear pulse propagation in the nanophotonic waveguide. The good agreement between the experiment and the GNLSE model is even conserved if only the free-carrier effects are included as perturbation to the soliton propagation, as presented in Fig. 2e,f. These results indicate FCD is the dominant perturbation and the cause of the soliton fission. We now perform additional GNLSE modelling to verify this observation and to examine the physical origin of the fission.

**Figure 3:**
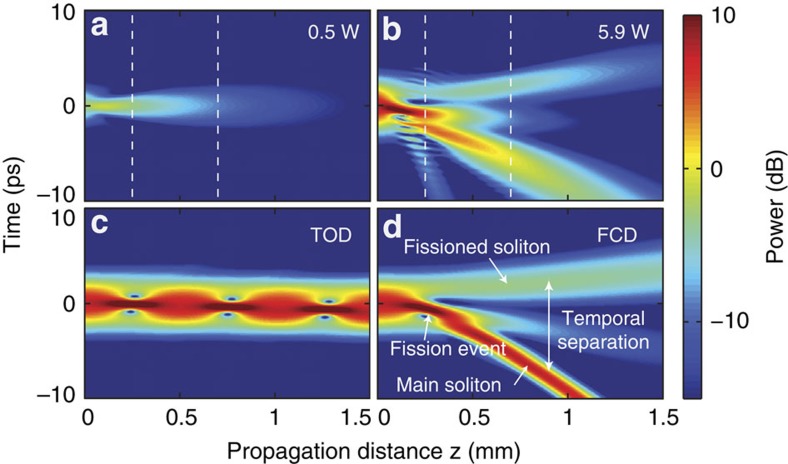
Time-space propagation maps from a generalised nonlinear Schrödinger equation model. (**a**–**d**) A GNLSE model of the pulse dynamics confirms the fission originates from free-carrier dispersion. The dashed lines indicate positions we measured along the waveguide. Note here we show the temporal power *P*(*t*) in a dB-scale relative to 1 W, whereas in Figs 1c and 2 we presented the cross-correlation of the electric field *E*(*t*), which is the quantity that we measure in the experiment. **a**,**b** correspond to the experimental conditions with low (**a**) and high (**b**) power, respectively. (**c**) The case modelled with solitons and a TOD perturbation. (**d**) Shows the case modelled with solitons, 3PA and a FCD perturbation.

Figure 3 summarizes our GNLSE modelling and confirmation that the fission event is triggered by FCD. In particular, we show the modelled pulse temporal *P*(*t*) profile along the waveguide. As a baseline, Fig. 3a,b show the GNLSE model in the linear (*P*_0_=0.5 W) and nonlinear (*P*_0_=5.9 W) regimes, respectively, with identical conditions to Fig. 2. The highest power level results in *L*_NL_=(*γP*_o_)^−1^=90 μm. The dashed white lines correspond to the two experimental spatial locations. We observe the pulses already split after ∼160 μm. We attribute the short fission length to a slow-light enhancement in the photonic crystal waveguide^18^. We now discern the roles of the different effects by switching them on and off independently in the model.

Figure 3c shows the case where we neglect free carriers by setting the carrier density *N_c_*=0 and include only TOD (*β_3_*) and the soliton terms (Kerr and GVD). The pulse clearly does not undergo fission but rather periodic recurrence as expected from soliton theory in the absence of perturbations^28^,^31^. This is not surprising due to the small relative magnitude of the TOD effect^3^. A similar result holds for SS. We conclude TOD and SS cannot be the fission mechanisms here. In contrast, Fig. 3d shows that setting *β*_3_=0 (including only FCD, 3PA and the soliton terms) yields a profile in which the main soliton advances in time with the smaller amplitude fissioned pulse trailing behind. The notable qualitative similarity of this result with both the full model (Fig. 3b) and the experimental result (Fig. 2d,f) confirms that FCD is the physical origin of the fission event. We note FCD scales as 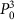, whereas 3PA scales as 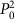, thus the reason for the strong FCD effect. Further, FCA is essentially negligible as shown by the ratio of the two effects 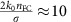. Now that we have established that FCD is the dominant perturbation in our system, we develop an analytic description to obtain deeper insight.

### Derivation of the free-carrier perturbation

It is common to write the GNLSE in a non-dimensional form to analyse the pulse evolution^27^. Here for our case of solitons for a free-carrier perturbation generated by 3PA this is:


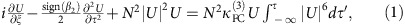


where ξ, 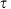 and *U* are the dimensionless parameters for propagation distance, time and pulse envelope, respectively (Supplementary Note 4). The terms on the left-hand side of the equation are related to soliton propagation. The right-hand side is reserved for perturbations, where the magnitude of the non-dimensional parameter governs the conditions to trigger soliton fission. A higher-order soliton will break apart when the magnitude of these parameters exceeds a minimum threshold. Conversely, when the parameter is below the threshold, the higher-order soliton remains intact and recurrent behaviour is retained. The numerical value of the minimum threshold depends on the specific physical effect causing the fission (that is, TOD, SS and FCD). An important additional property is that the minimum threshold to break a soliton decreases with increasing soliton number *N*, a topic we will treat in further detail below. Importantly, we have introduced the new term 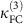 to elucidate the role of the FCD perturbation:


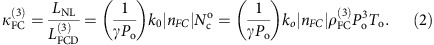


We have also defined 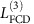, the FCD length for the free-carrier density generated dynamically from intrapulse 3PA with a peak carrier amplitude 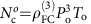, the free-carrier generation efficiency 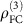 (ref. 11), and *T*_o_=*T*_FWHM_/1.76 for hyperbolic secant pulses. The physical interpretation of 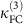 is the relative nonlinear phase shift due to the Kerr effect compared with FCD per unit length.

**Figure 4:**
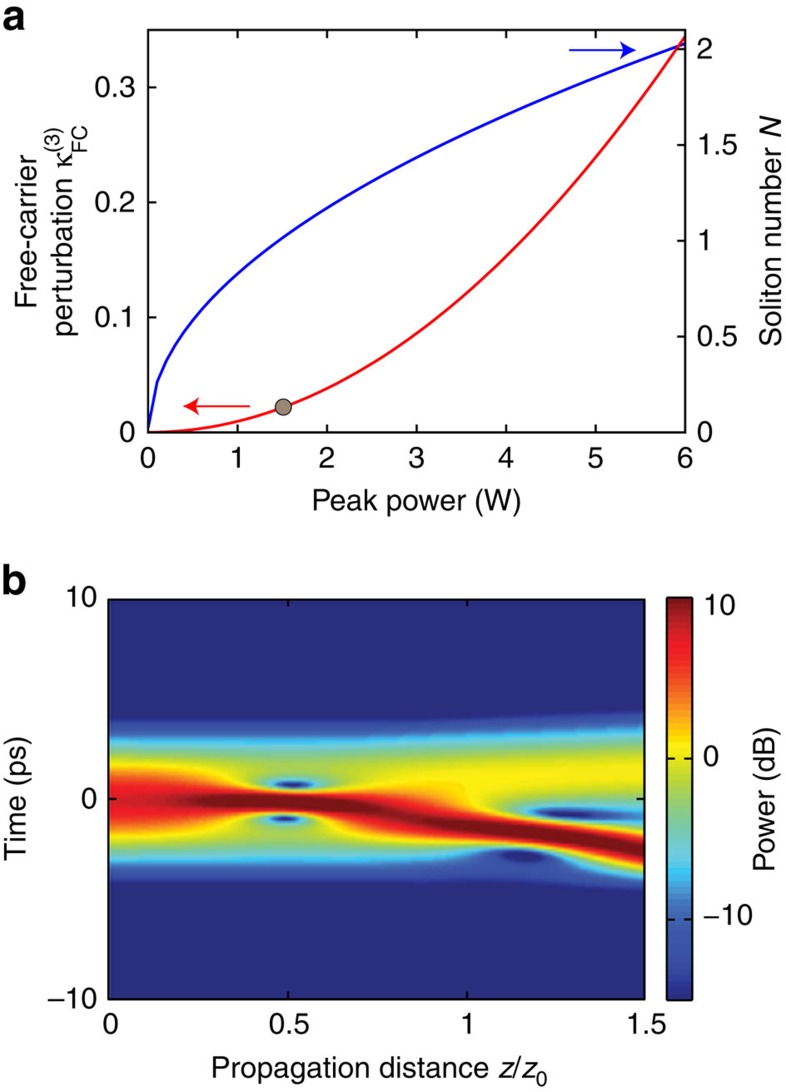
Analysis of the free-carrier perturbation generated from three-photon absorption. (**a**) Plot of the 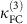 perturbation and the soliton number *N* versus power indicating the different scalings for each (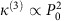 and 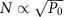). (**b**) GNLSE simulation showing the case with the minimum free-carrier dispersion perturbation 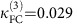 required for fission of a *N*=2 soliton. Note here we show the temporal power *P*(*t*) in a dB-scale relative to 1 W.

In terms of characteristic physical scaling, we see that 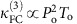, with the material contributing via constants. The power dependence comes from the nonlinear 3PA carrier generation, whereas the *T*_o_ term arises due to the fact that free carriers accumulate over the pulse duration, as represented by the integral in Equation (1). It is worth highlighting that the exact scaling of *κ*_FC_ depends on the specific nonlinear mechanism generating the free carriers (for example, two-photon absorption, ionized gas tunneling and so on). We describe this point further in the Discussion. Note that this carrier perturbation has a completely different form to perturbations caused by TOD, Raman and SS which scale as 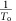 due to a derivative term 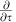 in the GNLSE, indicating these effects scale with the local pulse shape, rather than the non-local free-carrier effects^31^. Figure 4a shows the calculated 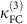 parameters as a function of coupled peak power for our experimental conditions. We have also included the scaling of the soliton number to show the comparative evolution of these two parameters (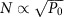 and 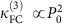).

### Analytic estimate of FCD-induced fission threshold

We now predict the minimum threshold of 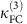 required to observe a fission event using this formalism and the characteristic scales for soliton period (*z*_*o*_) and time duration (*T*_o_) from the literature. First, we define the criteria to call an event a soliton fission. This is non-trivial as the fission is an adiabatic process characterized as a continuous spectral and temporal walk-off of the two constituent solitons^31^,^33^. We consequently define a clean fission to be a separation of *T*_0_ between the two pulses. Since even for the weakest FCD perturbation this separation can occur at very long distances, we further imposed the condition that the fission must occur within one soliton period *z*_0_ (ref. 3). Under these constraints for a soliton of order *N*=2 we derived an analytic threshold of 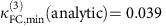 employing a moments method formalism and the equations in the text^29^. We show the full derivation in Supplementary Note 5. The maximum experimental value of 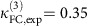 is approximately an order of magnitude larger than this minimum threshold and clearly of significant strength in the experiments. Note that one can choose arbitrary lengths, temporal separation and soliton number in these equations for desired experimental conditions (see equation 18 in Supplementary Note 5). For higher-order solitons one must consult the analytic relations for the constituent soliton powers following ref. 34 and substitute the appropriate values into the derived equations.

To confirm our analytic theory, we performed GNLSE simulations and varied the strength of 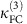. We did this by numerically reducing *n*_*FC*_ so as not to modify the relationship between the soliton number and 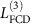. Figure 4b shows the simulation with 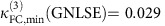, which is the minimum strength to meet our criteria. This is on the same order as the analytic theory with the difference attributed to momentum conservation from soliton recoil^4^. This is expected since our analytic formalism treats the constituents independently and neglects soliton interactions. Similar to the known behaviour for other perturbations, we found larger soliton numbers require smaller perturbations to break up the higher-order soliton^3^. This observation is supported by our analytic theory which shows 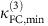 scales as 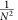 (see equation 21 in Supplementary Note 5). We compare the FCD perturbation strength derived here with known perturbation mechanisms such as TOD and SS in Supplementary Note 6.

## Discussion

From a fundamental physics perspective, these results apply to the general class of optical systems with nonlinear photocarrier (photoelectron) generation. Knowledge of the specific carrier generation mechanism is critical as the physical parameters governing the *κ*_FC_ perturbation scale differently in the tunneling and multiphoton ionization regimes^14^,^15^,^16^,^35^. For example, in the case of semiconductor waveguides, a related derivation of the plasma length 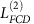 was shown for silicon (a TPA-limited material at our wavelength) though soliton perturbation was not addressed in that case^11^. We derive 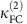 for TPA in Supplementary Note 7 and show that it scales linearly with power. Supplementary Figure 3 compares the power evolution of the 3PA and TPA cases. In the case of ionized gases, an equivalent plasma length for static ionized gases was provided in ref. 36 and we expect a *κ* parameter could be defined for dynamic nonlinear ionization based on the formalism in ref. 15 for the carrier generation term *N*_c_.

An important application of soliton fission is the generation of ultrabroad coherent light known as supercontinuum (SC)^31^,^37^,^38^,^39^. The demonstration of SC generation in photonic crystal fibres in 2000 (ref. 40) led to rapid adoption of SC sources in many fields including breakthrough experiments in metrology^41^, optical coherence tomography^42^ and optical frequency combs^43^. The utility of supercontinuum generation in fibre waveguides has led to significant interest in developing broadband light sources in integrated platforms^44^,^45^,^46^,^47^,^48^,^49^,^50^. Examining a recent investigation on supercontinuum in silicon, we computed a value of 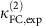 more than two orders of magnitude larger than our predicted threshold 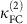
(analytic), indicating that the FCD perturbation is required to explain their results^48^ (Supplementary Note 7). We expect these observations will facilitate improved SC sources in integrated photonic chips envisioned for future on-chip optical communications systems^51^ and lab-on-a-chip spectroscopic tools^52^.

In summary, we demonstrated that free-carrier dispersion can induce soliton fission with both theoretical and experimental approaches. Our near-field microscopy measurements enabled the direct observation of the temporal and spatial evolution in the nanoscale waveguide, thereby providing a key new measurement technique for characterizing nonlinear pulses in sub-wavelength structures. We derived an analytic formulation and characteristic parameter 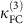 for the FCD perturbation and showed that our experimental values were an order of magnitude larger than the minimum required threshold. We supported these results with a GNLSE model confirming both the experiments and theory. These observations elucidate the fundamental physical scaling and dynamics of soliton fission in free-carrier media and could find applications in improved supercontinuum sources in integrated photonic chips and gas-filled microstructured fibres.

## Methods

### Sample description and material parameters

A scanning electron micrograph and detailed fabrication parameters of the *L*=1.5 mm air-suspended GaInP waveguide can be found in Supplementary Note 1 and Supplementary Table 1. Our sample includes integrated mode-adapters which reduce the total insertion losses in the linear regime to ∼17 dB (including propagation loss) and suppress Fabry–Perot oscillations at the end facets. The output coupling from the chip to the 0.4 numerical aperture (NA) lensed fibre (OzOptics) is −2.5 dB in agreement with our earlier work^17^. Due to the need to approach the NSOM tip near to the sample input, coupling is achieved with a 0.4 NA aspheric lens (Newport) with an estimated coupling efficiency of −7 dB which we suspect is due a mode-field size mismatch between the beam waist and the lens. We report the measurements of the sample properties, material parameters and the GNLSE model in Supplementary Notes 1 and 3.

### Experimental set-up

For the nonlinear measurements, we employed a mode-locked fibre laser (PriTel) delivering hyperbolic-secant pulses at 1,553 nm with a temporal duration *T*_FWHM_=2.2 ps as measured by autocorrelation. The repetition rate is 20 MHz and the laser light is coupled to the waveguide with electric-field polarized in-plane with the slab (TE). The pulses are slightly chirped as confirmed by autocorrelation measurements of the pulse input. For the nonlinear pulse transmission measurements, we used an optical spectrum analyser to measure the pulse spectrum as a function of input power. Two such traces are shown in Fig. 1a with additional traces in Supplementary Fig. 2.

To measure the temporal dynamics of the pulse propagating inside the waveguide we employ a homebuilt time-resolved NSOM^53^. In the set-up the entire microscope, including the sample, is included in one branch of a Mach–Zehnder interferometer. The near-field probe is brought in close proximity (circa 20 nm) of the waveguide where it collects the evanescent tail of the guided mode. As a result, a minute fraction of the guided light is transformed into far-field radiation by the near-field probe and is interferometrically mixed with light from a reference branch. The interference is detected on a photodiode with a heterodyne detection scheme. By scanning an optical delay line and using a pulsed laser source we measure a temporal cross-correlation of the electric field of the pulses propagating in the reference branch of the interferometer and in the waveguide. The measured temporal cross-correlation is described by the following equation:


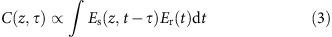


where 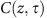 is the cross-correlation function, *z* the spatial location of the near-field probe, 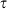 the delay time and 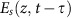 and *E*_*r*_(*t*) the electric field of the pulse propagating in the sample and the reference branch of the set-up, respectively. Correspondingly, the following equation holds in the frequency domain:


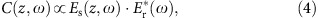


where *C*(*z*, *ω*), *E*_s_(*z*, *ω*) and E*_r_(*ω*) are the frequency spectra of the temporal cross-correlation function and the electric field of the pulse in the sample and the reference branch, respectively.

It has been shown that various changes of the temporal pulse envelope transfer to the cross-correlation function. For example, the time of flight can be directly extracted from the observed time delay in the experiment^54^. Furthermore, symmetric temporal broadening due to GVD^26^, as well as asymmetrical TOD^21^, exhibit similar features in the cross-correlation function as in the temporal pulse envelope. However, the cross-correlation function will only directly represent the temporal envelope of the pulse propagating in the sample if the pulse in the reference branch is extremely short in time, ideally a Dirac delta function, and its spectrum extremely broad and constant. Therefore, we show the temporal cross-correlation function in our manuscript where we discuss the experimental measurements (that is, in Figs 1c and 2).

To observe the nonlinear evolution of the pulse we repeat the cross-correlation measurements at different input powers which are controlled by a set of neutral density filtres. Further, to track the changes of the temporal pulse envelope in space we position the near-field probe at different locations along the waveguide and repeat the cross-correlation measurements. This measurement procedure allows, for example, to gain information of the time of flight of the pulse or the reshaping of the pulse envelope^21^. While there are a number of spatially resolved studies in the linear regime^21^,^55^, there are few investigations of nonlinear dynamics with NSOM^24^ or complementary techniques^25^ and, to our knowledge, no investigations of soliton dynamics.

## Additional information

**How to cite this article:** Husko, C. *et al*. Free-carrier induced soliton fission unveiled by *in situ* measurements in nanophotonic waveguides. *Nat. Commun.* 7:11332 doi: 10.1038/ncomms11332 (2016).

## Supplementary Material

Supplementary Information Supplementary Figures 1-3, Supplementary Table 1, Supplementary Notes 1-7 and Supplementary References.
