# Supplementary material for: Free-carrier-induced soliton fission unveiled by in situ measurements in nanophotonic waveguides
Source: Nat Commun. 2016 Apr 15;7:11332. doi: 10.1038/ncomms11332 (PMC4835551; doi:10.1038/ncomms11332)
Supplement: Supplementary Information — Supplementary Figures 1-3, Supplementary Table 1, Supplementary Notes 1-7 and Supplementary References. [file ncomms11332-s1.pdf]

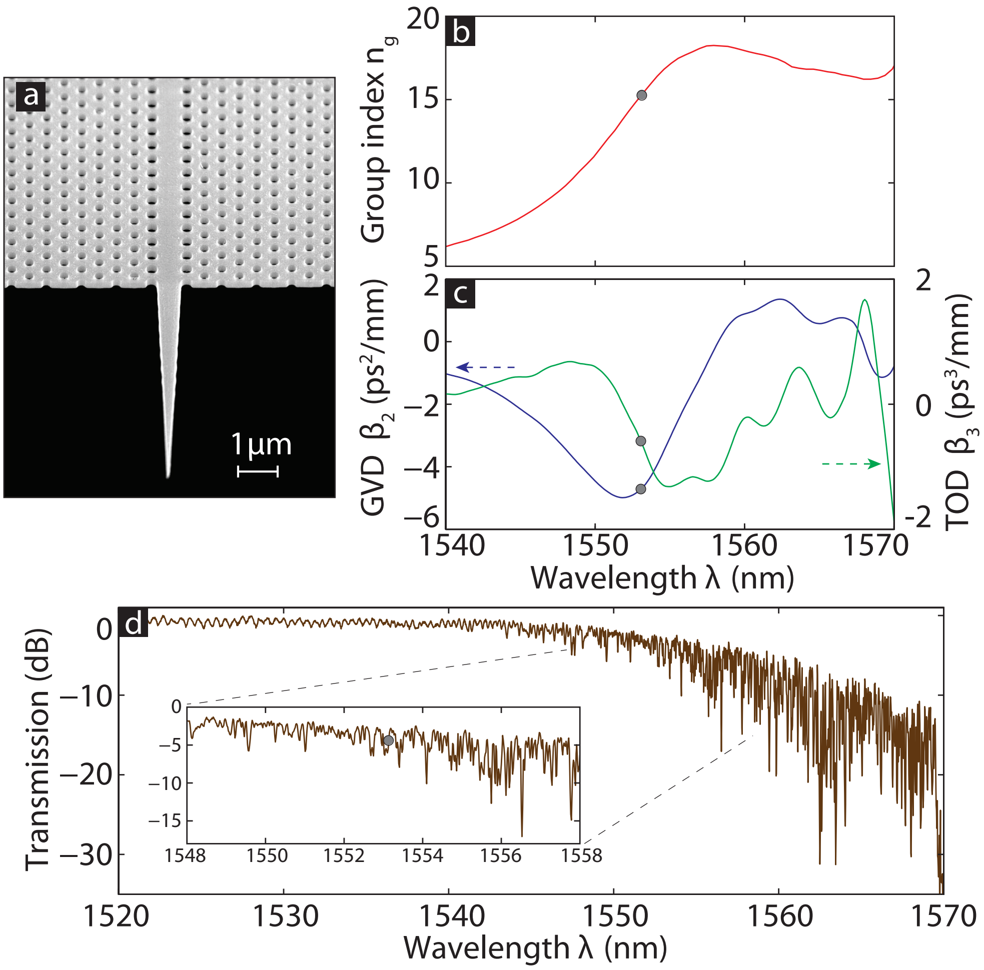

Supplementary Figure 1: Properties of the nanophotonic waveguide. (a) SEM image of the GaInP photonic crystal waveguide. (b) Measured group index versus wavelength show a slow-light dispersion engineered structure. (c) Group-velocity dispersion (GVD) and third-order dispersion (TOD) of the sample. (d) Measured linear transmission of the photonic crystal waveguide. Inset: zoom of the transmission in the wavelength region used in the experiments. The grey dot marks the wavelength where we performed our experiment.

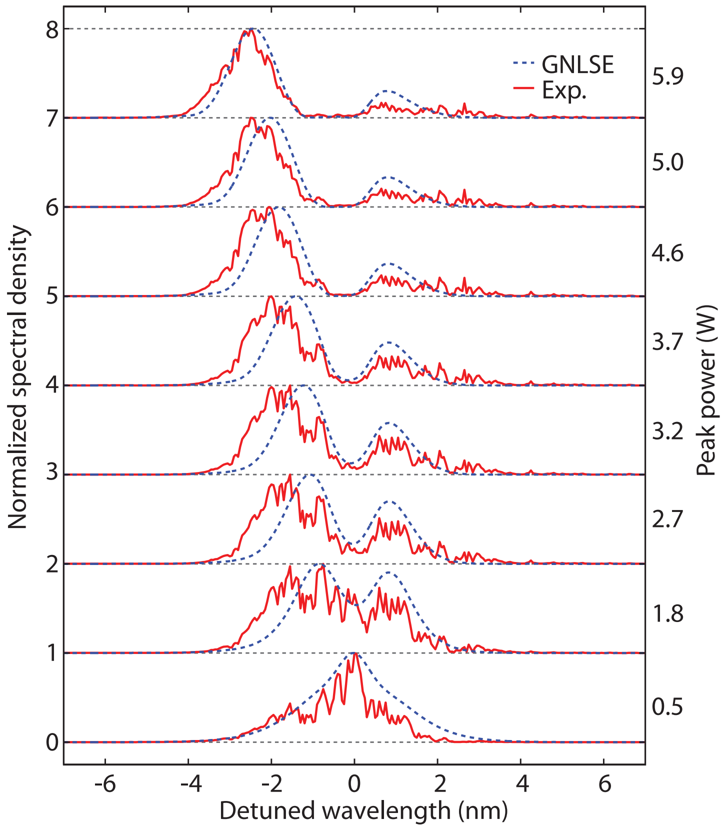

Supplementary Figure 2: Spectral measurements of the pulse transmission. Pulse transmission spectra measured with an optical spectrum analyser (solid) and generated from a GNLSE model (dashed). The vertical direction indicates increasing coupled peak power.

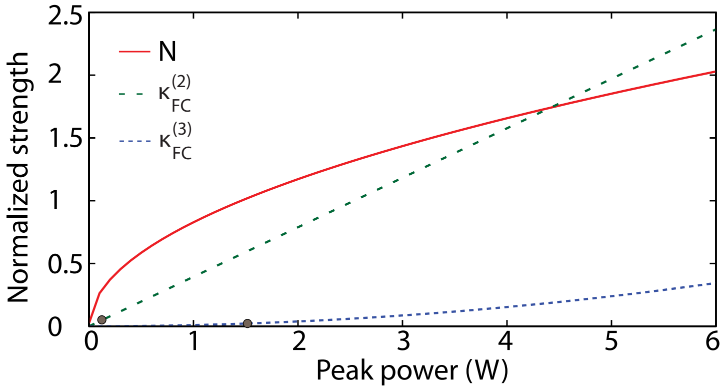

Supplementary Figure 3: Free-carrier dispersion perturbations for TPA and 3PA materials. Here we show the magnitudes of $\kappa_{FC}^{(2)}$ (Eqn. 23), $\kappa_{FC}^{(3)}$ (Eqn. 7) and the soliton number N versus peak power P_o. The markers indicate the minimum threshold from analytic modeling for the two effects.

Supplementary Table I: Semiconductor waveguide parameters at 1553 nm

α	40 dB/cm	L	1.5 mm
n_g	15.1	β_3	-0.55 ps^3/mm
β_2	-4.7 ps^2/mm	β_4	0.67 ps^4/mm
n_2	$7 \cdot 10^{-18} m^2 W^{-1}$	α_{3PA}	$1 \cdot 10^{-26} m^3 W^{-2}$
γ_{eff}	2100 $W^{-1}m^{-1}$	γ_{3PA}	20 $W^{-2}m^{-1}$
n_{FC}	$-3.7 \cdot 10^{-27}$ m^2	σ	$2.8 \cdot 10^{-21}$ m^2
η_{in}	-7 dB	η_{out}	-2.5 dB
A_{3eff}	0.31 μm^2	A_{5eff}	0.23 μm^2

Supplementary Note 1: Sample parameters

Figure 1 summarises the properties of the GaInP photonic crystal waveguide (PhCWG) used in the experiment. Figure 1(a) is an electron micrograph showing the air-holes in the air-clad dielectric slab with a line-defect that forms the waveguide section. The lattice period is $a = 471$ nm, with slab thickness 190 nm, and bulk hole radius $r = 0.23a$. The dispersion is engineered following our method outlined in Ref. [1]. Specifically, we tune the width of the defect to $W = 0.95\sqrt{3}a$, the innermost hole radii to $r_1 = 0.20a$ and the second row radii to $r_2 = 0.22a$. The innermost holes are also shifted outwards by $s_1 = 0.16\sqrt{3}/2a$. A twist parameter of $t = 0.1a$ yields a flat group index band. The GaInP semiconductor has a refractive index of n_0=3.17 and a band-gap of $E_g = 1.9$ eV. Regarding nonlinear properties, the leading effect is a $\chi^{(3)}$ electronic nonlinearity with a Kerr coefficient n_2 (intensity-dependent refractive index). At our working wavelength of 1553 nm ($\sim$0.8 eV) and the intensities in our experiment, we also observe three-photon absorption (3PA, α_3) in the wide-gap material [2].

Figures 1(b)-(c) show the dispersion properties of the fabricated device we measured using a homebuilt setup similar to that of Ref. [3]. Figure 1(b) is a plot of the group index n_g versus wavelength λ. Figure 1(c) shows the group-velocity dispersion (GVD, β_2) and third-order dispersion (TOD, β_3) coefficients of the device. Recall $\beta_n = d^n\beta(\omega)/d\omega^n$ is the nth-order dispersion of the propagation constant $\beta(\omega)$ at frequency ω. The GVD is anomalous, as required for sustaining solitons, and on the order of $\sim ps^2$/mm across the range of interest. The TOD term is small in the experiments, but included for completeness. Table I shows a summary of the experimental parameters described in the main text.

Supplementary Note 2: Spectral properties of the pulse transmission: experiment and model

Since soliton fission is fundamentally a time-domain phenomenon, we focused mostly on the experimentally measured temporal cross-correlations made with a brief discussion of spectral measurements. In this section we show the full measurement set of the spectral density of pulses transmitted through the PhCWG. Figure 2 shows the results of measurements (solid lines) made with an optical spectrum analyser. The vertical direction indicates increasing coupled peak power. The separation of the smaller second soliton is clearly visible as we increase the coupled power. As in the main text, we include the generalised nonlinear Schrödinger equation (GNLSE) model (dashed lines) with the solid agreement confirming the experimental results.

Supplementary Note 3: Generalised Nonlinear Schrödinger Equation Model (GNLSE)

The nonlinear pulse propagation in the GaInP semiconductor waveguide can be described by a GNLSE based on a similar approach as in Refs. [4, 5]. The following is the model we use throughout the main text. In addition to the dominant terms Kerr, GVD, and free-carrier dispersion (FCD, n_{FC}), we included higher-order terms in the full model in order to obtain a more accurate quantitative agreement between the GNLSE and the experimental data. These higher-order terms are: linear loss α, TOD, fourth-order dispersion (FOD, β_4), and self-steepening (SS, τ_{NL}). The inclusion of the higher-order terms also serves a didactic purpose here in the Supplementary Information as these perturbations are known in the literature and serve as a reference point to understand our current system. We emphasise the basic physics could be explained only with the soliton and FCD terms as shown by Figs. 2(e), 2(f) and 3(d) in the main text. The full model is:

$$i\frac{\partial A}{\partial z} - \frac{\beta_2}{2}\frac{\partial^2 A}{\partial t^2} + \gamma|A|^2 A = - i\frac{\alpha}{2}A + i\frac{\beta_3}{6}\frac{\partial^3 A}{\partial t^3} - \frac{\beta_4}{24}\frac{\partial^4 A}{\partial t^4} - \gamma\tau_{NL}\frac{\partial}{\partial t}|A|^2 A - i\frac{\alpha_{ThPA}}{2}|A|^4 A - (k_o n_{FC} + i\frac{\sigma}{2})N_c A. \quad (1)$$

On the left-hand side of the equation are the GVD and Kerr terms which compose the soliton while the right-hand side describes perturbations to the system. Here $A(z,t)$ is the slowly varying amplitude of the pulse, with time t in the frame of the pulse. The input pulse peak power is $P_0 = |A(0,0)|^2$ and k_0 is the vacuum wavenumber. The nonlinear self-phase modulation and 3PA terms are $\gamma = (k_0 n_2/A_{3eff})$ and $\alpha_{ThPA} = (\alpha_3/A_{5eff}^2)$, respectively, with the bulk coefficients for

n_2 [6] and α_3 [7]. The cross-sectional mode areas for the $\chi^{(3)}$ and $\chi^{(5)}$ nonlinear effects are A_{3eff} and A_{5eff}, respectively [8].

The FCD coefficient is n_{FC} with the free-carrier absorption cross-section (FCA, σ). Note that n_{FC} is a material parameter dependent on fundamental quantities and has a negative sign: $n_{FC} = (-e^2/(2n_0\epsilon_0\omega^2))\ (m_e^{*-1} + m_h^{*-1})$, where e is the elementary charge, ϵ_0 is the vacuum permittivity, ω is the angular frequency and $m^*_{e/h}$ is the effective mass of the electrons/holes in the semiconductor [9]. The free-carrier density $N_c(z,t)$ is controlled by the generation and recombination of the 3PA-induced free carriers according to the following rate equation:

$$\frac{\partial N_c}{\partial t} = \rho^{(3)}_{FC}|A|^6 - \frac{N_c}{\tau_c}, \tag{2}$$

where we have defined the power-normalised carrier generation rate $\rho^{(3)}_{FC} = \alpha_3/(3\hbar\omega_0 A^3_{5eff})$, with the photon energy $\hbar\omega_0$, and the effective free-carrier lifetime τ_c.

The self-steepening term τ_{NL} is related to the dispersion of $\gamma(\omega)$. Given the pulse duration T_{FWHM} =2.2 ps in our experiments, it is surprising that we include this term at all as it is typically reserved for ultrafast pulses of tens of femtoseconds [10]. Nonetheless, it has recently been shown that the slow-light contribution to $\gamma(\omega)$ requires a new definition of τ_{NL} and can add orders-of-magnitude to its strength [11]. In brief,

$$\tau_{NL} = \frac{1}{\gamma}\frac{\partial\gamma}{\partial\omega} = \frac{2}{n_g}\frac{\partial n_g}{\partial\omega} - \frac{1}{A_{eff}}\frac{\partial A_{eff}}{\partial\omega}. \tag{3}$$

Notice the traditional $\frac{1}{\omega}$ dependence is not included since it is around one hundred times smaller. The Raman effect is completely negligible in our system due to both its narrowband (≈100 GHz) and far-detuned frequency peak in semiconductor systems (10-15 THz) [12, 13].

As we conduct our experiments in a photonic crystal waveguide, we account for the reduced group velocity (increased group index n_g) and the associated slow-light enhancements to the path length and electric field by the slow down factor $S = v_p/v_g = n_g/n_0$ [14]. The GNLSE describes our experiment when we replace the bulk parameters with the appropriate scaled values. For the free-carrier terms, $n_{FC,eff} = S\cdot\ n_{FC}$, $\sigma_{eff} = S\cdot\sigma$, and for the nonlinear terms $\gamma_{eff} = S^2\cdot\ \gamma$[14] and $\alpha_{ThPA,eff} = S^3\cdot\ \alpha_{ThPA}$ [8]. This form is also applicable to channel waveguides with the appropriate S value.

Supplementary Note 4: Derivation of the free-carrier perturbation: three-photon absorption

It is common to non-dimensionalise the GNLSE (Eqn. 1) to analyse the relative strengths of physical effects governing the pulse evolution [15]. In the main text we introduced a new term

$\kappa_{FC}^{(3)}$ to describe the FCD perturbation using this procedure. With this non-dimensional parameter we analysed the strength of the perturbation in our experiments and found it to be an order-of-magnitude above the minimum threshold. In this section, we show the derivation of the $\kappa_{FC}^{(3)}$ term. In our formulation we ignore the loss terms (α, α_3, and σ) and FOD for clarity.

As we noted in the main text, these results are general to optical systems with nonlinear photo-carrier (photo-electron) generation including tunneling and multi-photon ionization regimes. Applying the specific carrier generation mechanism to the formalism that follows is critical as the physical parameters governing the κ perturbation will scale differently.

We substitute the following dimensionless variables into (Eqn. 1):

$$\xi = \frac{z}{L_D}, \quad \tau = \frac{t}{T_0}, \quad U(\xi,\tau) = A(z,t)/\sqrt{P_0}, \tag{4}$$

where ξ, τ, and U correspond to normalised propagation distance, time, and pulse envelope compared to the dispersion length L_D, pulse duration T_0, and peak power P_0, respectively. This gives:

$$i\frac{\partial U}{\partial \xi} - \frac{\text{sign}(\beta_2)}{2}\frac{\partial^2 U}{\partial \tau^2} + N^2|U|^2U = N^2\kappa_{FC}^{(3)}U\int_{-\infty}^{\tau}|U|^6 d\tau' + i\delta_3\frac{\partial^3 U}{\partial \tau^3} - is\frac{\partial(|U|^2U)}{\partial \tau}. \tag{5}$$

In arriving at this result, we have used the well-known definitions for the dispersion lengths L_D and L'_D, the nonlinear length L_{NL} and the soliton number N:

$$L_D = \frac{{T_0}^2}{|\beta_2|}, \quad L'_D = \frac{{T_0}^3}{|\beta_3|}, \quad L_{NL} = \frac{1}{\gamma P_0}, \quad N = \sqrt{\frac{L_D}{L_{NL}}}. \tag{6}$$

The non-dimensional parameters on the right-hand side govern the conditions to trigger soliton fission, amongst other effects [16, 17]. The parameters related to TOD ($\delta_3 = \frac{\beta_3}{6|\beta_2|T_o}$) [16] and SS ($s = \frac{\tau_{NL}}{T_o}$) [10] are well known. A higher-order soliton will break apart when the magnitude of any of these parameters exceeds a minimum threshold. Conversely, when the parameter is below the threshold, the higher-order soliton remains intact and recurrent behaviour is retained. The numerical value of the minimum threshold is different for each physical effect. Separate to this point, the minimum threshold to break a soliton decreases with increasing soliton number N. We will treat this topic in further detail below.

We now focus on the role of free carriers for which we have introduced the FCD perturbation $\kappa_{FC}^{(3)}$:

$$\kappa_{FC}^{(3)} = \frac{L_{NL}}{L_{FCD}^{(3)}} = \left(\frac{1}{\gamma P_o}\right) k_o|n_{FC}|\rho_{FC}^{(3)}P_o^3 T_o, \tag{7}$$

with the characteristic FCD-3PA length:

$$L^{(3)}_{FCD} = \frac{1}{k_0|n_{FC}|\rho^{(3)}_{FC}{P_0}^3T_0} = \frac{1}{k_0|n_{FC}|N^o_c}. \tag{8}$$

We have defined the free-carrier density generated from intrapulse 3PA with a peak amplitude $N^o_c = \rho^{(3)}_{FC}P^3_oT_o$. In order to derive the $L^{(3)}_{FCD}$ we solved the free-carrier generation equation (Eqn. 2) assuming negligible intrapulse recombination, along with the condition $n_{FC} < 0$ known from the Drude model [9]. The solution is $N_c(\xi,\tau) = \rho^{(3)}_{FC}{P_0}^3T_0\int_{-\infty}^{\tau}|U(\xi,\tau')|^6d\tau'$, where the integral can be solved for exact pulse shapes (i.e. hyperbolic secant or Gaussian).

Note $\kappa^{(3)}_{FC}$ depends on both the power and temporal duration of the input optical pulse, with the material contributing via constants. The FCD length is proportional to peak power cubed due to 3PA carrier generation. The pulse duration dependence comes from the accumulation of free carriers across the pulse, as represented by the integral in the carrier equation. Once again, different nonlinear carrier generation mechanisms will have different scaling. In contrast, the characteristic scale of the Raman effect depends only on the native *material* response τ_R [18]. More generally, the definition of L_{FCD} depends on the specific carrier-generation mechanism for N_c. For example, a related derivation was shown for materials limited by two-photon absorption (TPA, α_2) in [19] though the normalised perturbation was not analysed in that case. We show more details on this below due to the relevance in silicon waveguides. Now that we have our definitions in place, we treat these effects quantitatively to show that FCD is the dominant mechanism in our experiments.

Supplementary Note 5:
Analytic estimate of the minimum free-carrier perturbation strength to cause soliton fission

In the main text we presented both analytical and numerical methods to estimate a quantitative threshold $\kappa^{(3)}_{FC,min}$ for FCD to cause soliton fission. Here we show the analytic formalism and compare this with known parameters.

Using the method of moments, we can estimate the threshold for soliton fission to occur. This analysis is based on the general evolution equations described in Ref. [20]. The starting point is to consider the equations for the blueshift Ω and the temporal shift T_c due to FCD driven by 3PA:

$$\frac{d\Omega}{dz} = \frac{k_0|n_{FC}|}{E}\int_{-\infty}^{\infty}|A|^2\frac{\partial N_c}{\partial t}dt, \tag{9}$$

$$\frac{dT_c}{dz} = \beta_2\Omega. \tag{10}$$

with the pulse energy E and our other previously defined variables. The field envelope $A(z,t)$ is

such that the peak power is $P = |A|^2$. Neglecting recombination in the carrier generation equation (Eqn. 2) and inserting into Eqn. 9, we get the blueshift evolution:

$$\frac{d\Omega}{dz} = \frac{k_0|n_{FC}|\rho_{FC}^{(3)}}{E}\int_{-\infty}^{\infty}|A|^8 dt. \tag{11}$$

This equation will change depending on the specific carrier generation mechanism as noted earlier. We now use the hyperbolic secant pulse:

$$A(z,t) = \sqrt{\frac{E}{2\tau}}\text{sech}\left(\frac{t-T_c}{\tau}\right)\times \tag{12}$$

$$\exp\left(-i\Omega(t-T_c) - i\frac{C}{2\tau^2}(t-T_c)^2\right), \tag{13}$$

where τ is the pulse duration and C is the chirp. The blueshift evolution follows:

$$\frac{d\Omega}{dz} = \frac{16}{35}k_0|n_{FC}|\rho_{FC}^{(3)}\frac{E^3}{(2\tau)^3}. \tag{14}$$

Noting that for a hyperbolic secant pulse with peak power P the energy is $E = 2P\tau$, and that the chirp C is zero for solitons, we integrate over z assuming constant power to get:

$$\Omega = \frac{16}{35}k_0|n_{FC}|\rho_{FC}^{(3)}P^3. \tag{15}$$

The assumption of constant power is taken as a first approximation. Indeed, in our earlier work with the moments method we examined specific cases where one can solve for Ω and T_c analytically when power varies with distance due to, for example, dispersion, linear loss, or nonlinear loss. Critically, we found the input power P_o remains the key parameter and that any variation in the power along the waveguide becomes a modest scale factor without loss of generality to the main physics.

We can now evaluate the temporal shift due to the FCD blueshift. Substituting our result for Ω into Eqn. 10 and integrating in z assuming constant power gives:

$$T_c = \frac{8}{35}\beta_2 k_0|n_{FC}|\rho_{FC}^{(3)}P^3 z^2. \tag{16}$$

We can use this expression for T_c to estimate the threshold for FCD to cause soliton fission.

In order to proceed, we must first recall theory describing the amplitudes of the constituent solitons composing a more energetic higher-order soliton. The peak power P corresponds to a single constituent soliton fissioning out of the bound state [21]. We define the relative power $\eta = P/P_0$, where P_0 is the input peak power. For a $N = 2$ soliton, the constituent fundamental solitons

have relative powers $\eta = 9/4$ and $\eta = 1/4$ [22]. For higher-order solitons, one must consult these relations for the appropriate values. Considering a single constituent $P = \eta\ P_o$:

$$T_c = \eta^3 \frac{8}{35} \beta_2 k_0 |n_{FC}| \rho^{(3)}_{FC} P_0^3 z^2. \tag{17}$$

We normalise the temporal shift to the input pulse duration T_0 and use the definitions for $L^{(3)}_{FCD}$ and L_D above to get:

$$\frac{T_c}{T_0} = \eta^3 \frac{8}{35} \frac{z^2}{L_D L^{(3)}_{FCD}}. \tag{18}$$

At this point we employ the characteristic scales of solitons for length (z_o) and pulse duration (T_o) as in the main text. Namely, the shift must occur within a soliton period $z_0 = (\pi/2)L_D$. We also recall $\kappa^{(3)}_{FC} = L_{NL}/L^{(3)}_{FCD}$ and the soliton relation $L_D = N^2 L_{NL}$ to simplify to:

$$\left.\frac{T_c}{T_0}\right|_{z=z_0} = \eta^3 \frac{2\pi^2}{35} N^2 \kappa^{(3)}_{FC}. \tag{19}$$

Additionally, we set the fission threshold such that after one soliton period the main pulse has advanced by T_0, i.e. $T_c/T_0 = 1$. Inverting this relation we get the main result:

$$\kappa^{(3)}_{FC,min}(analytic) > \left(\eta^3 \frac{2\pi^2}{35} N^2\right)^{-1}. \tag{20}$$

Importantly, $\kappa^{(3)}_{FC,min}(analytic) \propto \frac{1}{N^2}$, which explains why solitons of higher order N are easier to break apart.

Considering the highest power constituent of a $N = 2$ soliton has an amplitude of $\eta = 9/4$, we get the quantitative threshold for the N=2 soliton:

$$\kappa^{(3)}_{FC,min}(analytic) > \left((9/4)^3 \frac{2\pi^2}{35} N^2\right)^{-1} \tag{21}$$

$$\kappa^{(3)}_{FC,min}(analytic) > 0.039. \tag{22}$$

At the maximum power in our experiments we had $\kappa^{(3)}_{FC,max}$=0.35, which is an order-of-magnitude above the minimum threshold $\kappa^{(3)}_{FC,min}$ predicted by both analytic theory and simulation. This value is about 50% larger than the value obtained from numerical simulations $\kappa^{(3)}_{FC,min}$(GNLSE) = 0.029. The soliton recoil from momentum conservation probably accounts for most of this deviation [21]. This is expected since our analytic formalism treats the constituents independently and neglects soliton interactions. Indeed, for a fission of a $N = 2$ soliton caused by the Raman effect, the discrepancy between the threshold predicted by analytic theory and simulations was about 2.5, however after a much longer propagation distance of $\approx 6z_0$. The smaller soliton has amplitude η=1/4 and its temporal shift is negligible.

Supplementary Note 6: Comparison with known perturbation mechanisms

As mentioned above, the TOD perturbation δ_3 is well known. It has been shown numerically that a value of $\delta_{3,min} = 0.022$ can trigger fission for a soliton of order $N = 2$ [16]. For our parameters $\delta_3 = 0.016$, which is below the threshold. Figure 3(c) of the main text shows the higher-order soliton under the influence of TOD only. The N=2 soliton evolves periodically with no visible temporal separation. In contrast, Fig. 3(d) of the main text shows clear temporal separation of the solitons under the influence of FCD, highlighting that TOD is much weaker than FCD for our experimental conditions.

For SS, we use the formalism in Ref. [11] which includes contributions from the waveguide dispersion, slow light, and modulation in the mode area. Since we could not find a minimum threshold s_{min} in the literature, we conducted simulations and found the threshold to be s_{min}=0.17. Using the GNLSE we found our experimental value of $s = \frac{\tau_{NL}}{T_0}$ =0.09 (including the contributions from dispersion and area) is not sufficient to trigger fission and gives qualitatively similar results to Fig. 3(c) in the main text. This is not surprising as it is a factor of two smaller than the threshold.

In summary, the FCD perturbation $\kappa_{FC}^{(3)}$ in our experiments was ten times larger than the minimum required threshold $\kappa_{FC,min}^{(3)}$ and is moreover the strongest perturbation to the higher-order soliton propagation. We thus conclude the FCD is the dominant fission mechanism in our experiments.

Supplementary Note 7: Analytic treatment of fission from free carriers generated by TPA

Given the strong interest in the optical properties of silicon (E_g=1.1 eV) at 1550 nm, we also quote the equivalent threshold for soliton fission caused by free carriers generated from two-photon absorption. Following a similar procedure as above we find:

$$\kappa_{FC}^{(2)} = \frac{L_{NL}}{L_{FCD}^{(2)}} = \left(\frac{1}{\gamma P_o}\right) k_o |n_{FC}| \rho_{FC}^{(2)} P_o^2 T_o, \tag{23}$$

with the appropriate FCD length from TPA as in Ref. [19]:

$$L_{FCD}^{(2)} = \frac{1}{k_0 |n_{FC}| \rho_{FC}^{(2)} {P_0}^2 T_0}. \tag{24}$$

Once again we define the power-normalised free-carrier generation rate $\rho_{FC}^{(2)} = \alpha_2/(2\hbar\omega_0 A_{3eff}^2)$. Note that the absolute magnitude of $\kappa_{FC}^{(2)} \propto P_o T_o$ whereas $\kappa_{FC}^{(3)} \propto P_o^2 T_o$.

Using the moments method treatment above and the results for Ω and T_c in Ref. [20] we find the minimum threshold:

$$\kappa^{(2)}_{FC,min}(analytic) > \left((9/4)^2 \frac{\pi^2}{15} N^2\right)^{-1} \quad (25)$$

$$\kappa^{(2)}_{FC,min}(analytic) > 0.075 \quad (26)$$

This value is about a factor of two larger than the 3PA parameter. Importantly, the non-dimensional threshold still scales as $\frac{1}{N^2}$.

Figure 3 shows a comparison of $\kappa^{(2)}_{FC}$ and $\kappa^{(3)}_{FC}$ for identical waveguide parameters used in this study. The TPA coefficient for silicon at 1550 nm is taken as its experimentally measured value of $\alpha_2 = 1$ cm/GW [23]. Note the TPA perturbation $\kappa^{(2)}_{FC}$ grows both linearly in power and much more rapidly than the 3PA perturbation. We also highlight the minimum thresholds with markers. The TPA material clearly exceeds the threshold at a peak power that is smaller by an order-of-magnitude, highlighting the stronger relative impact of free carriers in the TPA system.

In the main text, we highlighted the importance of the FCD fission mechanism in explaining recent supercontinuum (SC) generation experiments in silicon [24]. Recall that it has been shown that the larger the soliton order N, the smaller the required perturbation to cause soliton fission [16]. In. Ref. [24], they have N=19, and $\kappa^{(2)}_{FC,exp} \approx$ 2.4, which exceeds the N=2 threshold by more than a factor of thirty, and at least two orders of magnitude larger than the fission threshold for a N=19 soliton. It is clear that FCD played a significant role in that experiment. Though not measured, it is likely the fissioned solitons would have advanced in time in the semiconductor medium, and the broadening on the blue side attributed to blue-shifting solitons. We suggest all past, present, and future supercontinuum demonstrations in semiconductor waveguides and ionized gases consider the role of FCD in order to create improved on-chip integrated SC sources with broader and brighter spectra.

Supplementary References

[1] Colman, P., Combrié, S., Lehoucq, G. & De Rossi, A. Control of dispersion in photonic crystal waveguides using group symmetry theory. *Optics Express* **20**, 13108–13114 (2012).

[2] Combrié, Sylvain and Tran, Quynh Vy and De Rossi, Alfredo and Husko, Chad and Colman, Pierre. High quality GaInP nonlinear photonic crystals with minimized nonlinear absorption. *Applied Physics Letters* **95**, 221108–221108 (2009).

[3] Soller, B., Gifford, D., Wolfe, M. & Froggatt, M. High resolution optical frequency domain reflectometry for characterization of components and assemblies. *Optics Express* **13**, 666–674 (2005).

[4] Husko, C. A. *et al.* Soliton dynamics in the multiphoton plasma regime. *Sci. Rep.* **3**, 1100 (2013).

[5] Colman, P. *et al.* Temporal solitons and pulse compression in photonic crystal waveguides. *Nature Photonics* **4**, 862–868 (2010).

[6] Sheik-Bahae, M., Hagan, D. & Van Stryland, E. W. Dispersion and band-gap scaling of the electronic Kerr effect in solids associated with two-photon absorption. *Phys. Rev. Lett.* **65**, 96 (1990).

[7] Wherrett, B. S. Scaling rules for multiphoton interband absorption in semiconductors. *JOSA B* **1**, 67–72 (1984).

[8] Husko, Chad and Combrié, Sylvain and Tran, Quynh V and Raineri, Fabrice and Wong, Chee Wei and De Rossi, Alfredo. Non-trivial scaling of self-phase modulation and three-photon absorption in III-V photonic crystal waveguides. *Optics Express* **17**, 22442–22451 (2009).

[9] Soref, R. A. & Bennett, B. R. Electrooptical effects in silicon. *Quantum Electronics, IEEE Journal of* **23**, 123–129 (1987).

[10] Anderson, D. & Lisak, M. Nonlinear asymmetric self-phase modulation and self-steepening of pulses in long optical waveguides. *Physical Review A* **27**, 1393 (1983).

[11] Husko, C. & Colman, P. Giant anomalous self-steepening in photonic crystal waveguides. *Phys. Rev. A* **92**, 013816 (2015).

[12] Lee, H. *et al.* Study of strain and disorder of $In_xGa_{1-x}P$/(GaAs, graded GaP)($0.25 \leq x \leq 0.8$) using spectroscopic ellipsometry and Raman spectroscopy. *Journal of Applied Physics* **75**, 5040–5051 (1994).

[13] Boyraz, O. & Jalali, B. Demonstration of a silicon raman laser. *Optics Express* **12**, 5269–5273 (2004).

[14] Bhat, N. A. R. & Sipe, J. E. Optical pulse propagation in nonlinear photonic crystals. *Physical Review E* **64**, 056604 (2001).

[15] Agrawal, G. P. *Nonlinear Fiber Optics* (Academic Press, 5th ed., 2013).

[16] Wai, P. K. A., Menyuk, C. R., Lee, Y. C. & Chen, H. H. Nonlinear pulse propagation in the neighborhood of the zero-dispersion wavelength of monomode optical fibers. *Optics Letters* **11**, 464–466 (1986).

[17] Dudley, J. M., Genty, G. & Coen, S. Supercontinuum generation in photonic crystal fiber. *Rev. Mod.*

Phys. **78**, 1135–1184 (2006).

[18] Gordon, J. P. Theory of the soliton self-frequency shift. *Optics Letters* **11**, 662–664 (1986).

[19] Blanco-Redondo, A. *et al.* Controlling free-carrier temporal effects in silicon by dispersion engineering. *Optica* **1**, 299–306 (2014).

[20] Lefrancois, S., Husko, C., Blanco-Redondo, A. & Eggleton, B. J. Nonlinear silicon photonics analyzed with the moment method. *JOSA B* **32**, 218–226 (2015).

[21] Tai, K., Bekki, N. & Hasegawa, A. Fission of optical solitons induced by stimulated Raman effect. *Optics Letters* **13**, 392–394 (1988).

[22] Satsuma, J. & Yajima, N. B. Initial Value Problems of One-Dimensional Self-Modulation of Nonlinear Waves in Dispersive Media. *Progress of Theoretical Physics Supplement* **55**, 284–306 (1974).

[23] Bristow, A. D., Rotenberg, N. & Van Driel, H. M. Two-photon absorption and Kerr coefficients of silicon for 850–2200 nm. *Applied Physics Letters* **90**, 191104–191104 (2007).

[24] Leo, F. *et al.* Dispersive wave emission and supercontinuum generation in a silicon wire waveguide pumped around the 1550 nm telecommunication wavelength. *Optics Letters* **39**, 3623–3626 (2014).
